# Real-Time Real-World Digital Monitoring of Adolescent Suicide Risk During the Six Months Following Emergency Department Discharge: Protocol for an Intensive Longitudinal Study

**DOI:** 10.2196/46464

**Published:** 2023-06-26

**Authors:** Shira Barzilay, Shai Fine, Shannel Akhavan, Liat Haruvi-Catalan, Alan Apter, Anat Brunstein-Klomek, Lior Carmi, Mishael Zohar, Inbar Kinarty, Talia Friedman, Silvana Fennig

**Affiliations:** 1 Department of Community Mental Health University of Haifa Haifa Israel; 2 Schneider Children's Medical Center of Israel Petach Tikva Israel; 3 Data Science Institute Reichman University Herzliya Israel; 4 Ivcher School of Psychology Reichman University Herzliya Israel

**Keywords:** suicide, suicide ideation, suicide prevention, adolescents, real-time assessment, digital phenotyping, risk assessment, mobile phone

## Abstract

**Background:**

Suicide is the second leading cause of death in adolescents, and self-harm is one of the strongest predictors of death by suicide. The rates of adolescents presenting to emergency departments (EDs) for suicidal thoughts and behaviors (STBs) have increased. Still, existing follow-up after ED discharge is inadequate, leaving a high-risk period for reattempts and suicide. There is a need for innovative evaluation of imminent suicide risk factors in these patients, focusing on continuous real-time evaluations with low assessment burden and minimal reliance on patient disclosure of suicidal intent.

**Objective:**

This study examines prospective longitudinal associations between observed real-time mobile passive sensing, including communication and activity patterns, and clinical and self-reported assessments of STB over 6 months.

**Methods:**

This study will include 90 adolescents recruited on their first outpatient clinic visit following their discharge from the ED due to a recent STB. Participants will complete brief weekly assessments and be monitored continuously for their mobile app usage, including mobility, activity, and communication patterns, over 6 months using the iFeel research app. Participants will complete 4 in-person visits for clinical assessment at baseline and at the 1-, 3-, and 6-month follow-ups. The digital data will be processed, involving feature extraction, scaling, selection, and dimensionality reduction. Passive monitoring data will be analyzed using both classical machine learning models and deep learning models to identify proximal associations between real-time observed communication, activity patterns, and STB. The data will be split into a training and validation data set, and predictions will be matched against the clinical evaluations and self-reported STB events (ie, labels). To use both labeled and unlabeled digital data (ie, passively collected), we will use semisupervised methods in conjunction with a novel method that is based on anomaly detection notions.

**Results:**

Participant recruitment and follow-up started in February 2021 and are expected to be completed by 2024. We expect to find prospective proximal associations between mobile sensor communication, activity data, and STB outcomes. We will test predictive models for suicidal behaviors among high-risk adolescents.

**Conclusions:**

Developing digital markers of STB in a real-world sample of high-risk adolescents presenting to ED can inform different interventions and provide an objective means to assess the risk of suicidal behaviors. The results of this study will be the first step toward large-scale validation that may lead to suicide risk measures that aid psychiatric follow-up, decision-making, and targeted treatments. This novel assessment could facilitate timely identification and intervention to save young people’s lives.

**International Registered Report Identifier (IRRID):**

DERR1-10.2196/46464

## Introduction

### Background

Suicide is the second-leading cause of death globally among adolescents. Adolescence is a critically vulnerable developmental period for suicidal thoughts and behaviors (STBs). Youth emergency department (ED) visits for STB increased in many countries, particularly during the COVID-19 pandemic [1,2]. These incidents extend into long-term risk, as a history of suicide behaviors among youth is associated with an increased risk of repeat attempts and subsequent death [3]. The weeks and months following ED admission are at the highest risk for reattempts and suicide [4]. Suicidal crises in adolescents are often brief and episodic, with considerable potential for recurrence [5]. Adolescents are susceptible to interpersonal belonging, rejection, and conflict that may predispose them to imminent STB [6]. They have limited ability to regulate emotions and use cognitive control to inhibit problem behaviors, including suicidal acts when facing acute stress [5]. Therefore, the rapid-fluctuating acute suicidal states in adolescents warrant an improved proximal prediction of imminent STB.

Identifying proximal predictors for STB is critical to a better understanding of when individuals are most at risk [7]. It is distinguished from the unsuccessful scientific and clinical attempts to predict who is at risk [8] at the population level. In other words, the critical problem is to predict whether an individual with well-known preexisting risk factors is at imminent suicide risk (ie, within the next hours, days, or weeks) [9]. Two presuicidal mental state-specific diagnoses have been recently proposed in this context. Acute affective disturbance and suicide crisis syndrome are 2 conditions suggested to lead to imminent suicidal thoughts or behaviors [10,11]. Acute suicidal affective disturbance [12] is characterized by a sudden increase in suicidal intent and marked social alienation. Suicide crisis syndrome [13] is characterized by an intense sense of being trapped or unable to see any other way out. This condition is accompanied by experiencing affective disturbance, loss of cognitive control, and difficulty functioning in their social lives. The transdiagnostic approach suggested by these 2 diagnoses not only enables a more accurate and objective assessment of imminent suicide risk but could also facilitate research in developing digital markers of suicide risk.

Intensive longitudinal methods (ie, repeatedly assessing individuals over time) provide the unique opportunity to study proximal predictors of STB within individuals. Recent studies have begun using real-time mobile and wearable devices to predict STB, and the feasibility and acceptability of intensive monitoring in adolescents have been established [14,15]. Existing efforts are mainly focused on using intensive self-report assessments such as ecological momentary and daily diaries. These studies provided valuable information about the temporal dynamics of suicidal ideation [16,17] and significant proximal predictors, such as sleep problems and interpersonal stress [18-20]. However, the substantial reliance on self-reports requires insight and compliance, and the associated assessment burden limits study durations and applicability to real-world settings. Passive sensing of mobile data has been proposed as a promising direction for future research [14,21]. It allows naturalistic and continuous data collection with minimal burden, including social, communication, activity, and sleep patterns [22], in which change may indicate imminent STB. Recognizing objective web-based and offline social behavior patterns preceding STB may be particularly beneficial for adolescents. Pioneering studies have demonstrated that passive mobile sensing by actigraphy [23-26] may predict STB. An existing study demonstrated the usefulness of predicting symptoms in patients with major depressive disorder through passive data collection (electrodermal activity, sleep patterns, motion, communication, location changes, and phone usage patterns) from built-in sensors in phones and a wearable device [23-26]. Another study demonstrated the feasibility of predicting patients’ moods using machine learning algorithms with privacy-preserving techniques through multimodal data collection (text, audio, and sensor data) from patients’ mobile devices [23-26]. However, to our knowledge, there are no studies investigating proximal predictors of youth STB via real-time mobile passive sensing, including communication and activity patterns, and clinical and self-reported assessments over 6 months.

Unlike previous research that considered individual risk factors, a machine learning approach to passive mobile sensing can examine multiple risk factors and their combinations to build superior prediction models. This approach may identify complex real-life patterns and relationships and improve the near-term identification of risk and timely interventions [27,28]. However, the performance of passive sensing and machine learning over traditional methods for suicide risk classification and the interpretability of their findings demand further research [29-31]. Additional work in naturalistic settings is needed to unlock the potential of active and passive sensing and machine learning prediction models for suicide prevention. In addition, further research is required to develop prediction models optimized for implementation in clinical settings [28-30,32,33]. A longer follow-up period is needed to examine proximal predictors of suicidal behaviors (ie, recurrent attempts, ED admissions, and hospitalization) that may be clinically informative for suicide risk management in clinical settings. Furthermore, previous studies typically involved substantial payments for study participation, intensive compliance strategies, and safety procedures that may not be generalizable to real-world settings. This study addresses these challenges and will use intensive digital monitoring of self-reported and behavioral data in a real-time and real-world outpatient clinical setting.

### Study Aims and Objectives

#### Overview

This study will address the knowledge gap in real-time proximal predictors of youth STB. It will be the first to use active and passive digital monitoring in a longitudinal prospective study among high-risk adolescents in a real-world clinical setting. The current research, therefore, attempts to provide a proof of concept for digital markers of imminent STB to classify adolescents with high-risk STB versus those with lower risk, enabling a more accurate and objective assessment of imminent suicide risk. The project’s objectives integrate multiple digitally derived data sources, including behavioral, interpersonal, and frequent subjective self-reports, using comprehensive and nonintrusive techniques. This project has 2 main aims.

#### Aim 1

The first aim is to demonstrate the clinical utility of digital weekly self-reports in the identification of adolescents at risk for STB in a real-world high-risk clinical setting.

- Hypothesis 1: There will be satisfactory compliance (>60%) for weekly digital assessments of STB and main risk factors, and it will be accepted among adolescents, parents, and mental health professionals for routine clinical suicide risk management and care.
- Hypothesis 2: The weekly digital assessments of STB and main risk factors will demonstrate concurrent and prospective associations with clinical assessments of STB at the 1-, 3-, and 6-month follow-up, measured by the Columbia Suicide Severity Scale (C-SSRS) [34], and ED admissions.

#### Aim 2

The second aim is to develop an integrated prediction model using passive mobile sensing data for STB prediction.

- Hypothesis 3: The passively derived mobile usage data will accurately classify adolescents with high-risk STB versus those with lower risk over 6 months of follow-up as determined by clinical evaluations.
- Hypothesis 4: The passively derived mobile usage data will predict same- and next-week adolescents’ self-reported STB.
- Hypothesis 5: Integrating passive sensing and active self-reports will predict high-risk STB more accurately than each data source alone.

Overall, this project aims to use state-of-the-art digital technologies to identify proximal predictors of youth STB. These methods may provide the invaluable capacity to develop real-time prediction models, crucial steps toward clinical applications, and life-saving interventions.

## Methods

### Study Design

The study target sample includes 90 high-risk adolescents aged 11-18 years presenting to the ED with recent STB and recruited at the Depression and Self-Harm Clinic at Schneider Children’s Medical Center of Israel. All eligible patients identified in the ED will be referred to the Depression and Self-Harm Clinic. Participants will complete brief weekly assessments and be monitored continuously for their mobile app usage, including mobility, activity, and communication patterns, over 6 months using the iFeel (inManage Ltd) research app. Participants will complete 4 in-person visits for clinical assessment at baseline and at the 1-, 3-, and 6-month follow-ups.

### Procedures

Consecutive admissions to the clinic will be recruited for the study. Clinical staff will notify the research staff of potential eligible recruitment. Senior research staff will explain the study in detail according to local ethical requirements, followed by signed parental consent and the child’s signed assent (if over 16 years). Study participation, or lack thereof, will not affect the standard psychosocial and pharmacological services provided to participants. The response rate and time between ED admission and clinic admission will be recorded and examined in this study.

The inclusion criteria includes (1) adolescents between the ages of 11 and 18 years presenting with recent (past month) suicidal ideation or suicidal behavior (3 months), as defined by the C-SSRS [34] (preparatory acts, aborted, interrupted, and actual suicide attempts, or nonsuicidal self-injury), and (2) Android phone users (required for the iFeel app).

The exclusion criteria includes participants with acute medical conditions, mental retardation, cognitive impairment, or linguistic limitations preclude understanding the research questions.

**Figure 1:**
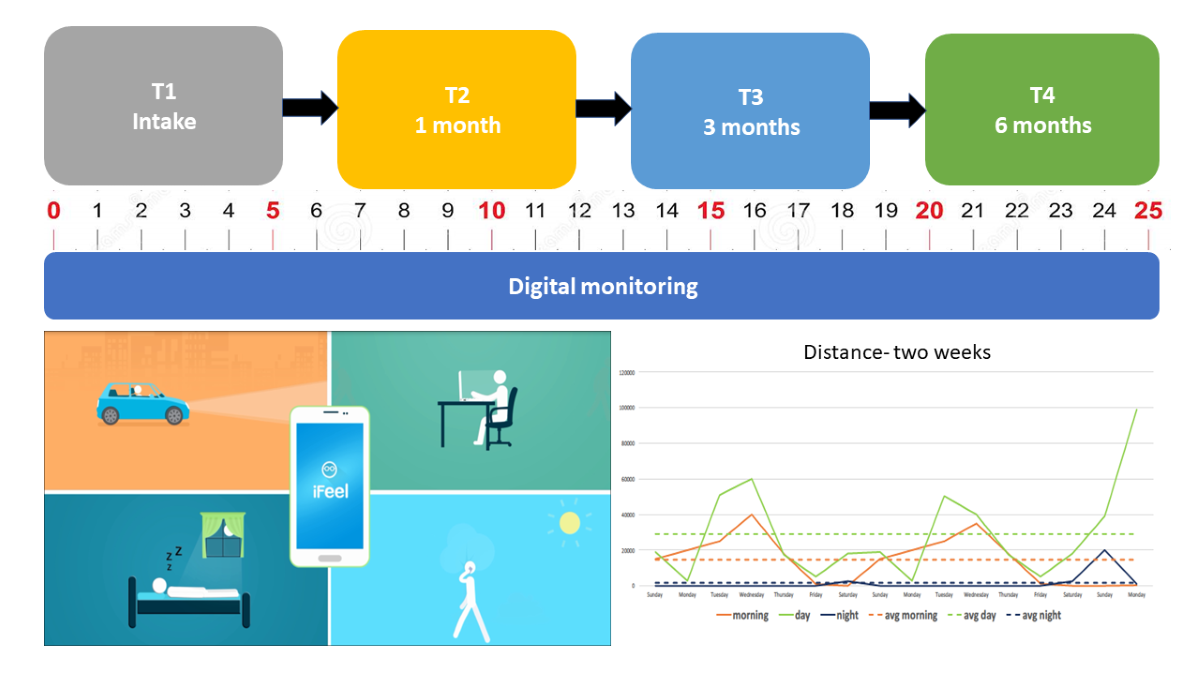
Illustration of study assessments. Initial and 1-, 3-, and 6-month extended assessments; 25 weekly digital self-report assessments over 6 months; and continuous passive mobile data usage monitoring (including mobility, activity, communication, and sleep patterns).

Participants will complete a comprehensive assessment battery (T1) and download the iFeel app using a random app-generated code. Once downloaded, the app will gather relevant information from the mobile phone for the next 6 months and prompt participants to respond to weekly brief questionnaires, including STB measurements and main risk factors. Research assistants will train the participants on the weekly survey protocol and questions, emphasize the importance of adherence, provide examples, and instruct them on how to use the subjective rating scales. Research assistants will contact participants during the follow-up period in cases of missing active or passive data collection and assist with technical or adherence issues. Participants will be invited for extended follow-up evaluations at 1 (T2), 3 (T3), and 6 months following the initial assessment (T4; see Figure 1). These will include in-person or remote video clinical assessments and self-report assessment scales administered via secured web-based surveys.

### Real-Time Mobile Data Collection via the iFeel App

iFeel is an innovative digital health research platform that enables passive and active digital monitoring and provides continuous objective measurements for any given disorder. iFeel will harness mobile phone data to find associations between digital markers and STB ratings obtained via weekly self-reports and clinical assessments during study visits. The data will be collected using the iFeel mobile phone app. The information to be measured via mobile phones using the app will consist of the following:

- Communication patterns: number of phone calls (without phone numbers), duration of incoming and outgoing phone calls, communication app usage duration
- Activity patterns: accelerometer data and total distance accumulated
- Device usage: number of devices and screen opens and locks, time of screen active, Wi-Fi connections, Bluetooth connections, data usage (bits), battery usage, log of app usage, luminosity, device positioning (horizontal or vertical), and screen touch parameters (strength and frequency of touch)

All the collected information has no identifiable information and no intention to change the patient’s treatment in this study.

### Weekly Data Collection for STB

A brief self-report questionnaire will be generated by the iFeel app using push notifications once a week. Three items were selected to assess the main risk factors for STB based on the presuicidal mental state–specific diagnoses (ie, acute suicidal affective disorder and suicide crisis syndrome). These include general mood (“In general, how was your mood this week?”), entrapment (“Did you feel like there was no way out?”), and social isolation (“Did you feel isolated?”), rated on a visual analog scale between 1 and 5. The STB items are adapted from the C-SSRS [34] and include assessing suicidal ideation intensity (rated on a visual analog scale between 1 and 5), intent, plan, behavior, attempt, and nonsuicidal self-injury. If a participant indicates yes to a suicidal plan, current intent, or behavior, a safety protocol will be initiated whereby crisis and community services are made available to the adolescent, and contact is made with a parent (see the Ethics Approval and Adverse Events and Safety Procedures section).

### In-Person Visits Measures (T1-T4)

The in-person visits measures include the following:

1. The Mini International Neuropsychiatric Interview for Children and Adolescents (MINI-KID) [35] will be administered at the initial assessment.
2. C-SSRS (Child Version) [34]: Evidence-based and commonly used suicidality scale.
3. Youth Suicide Crisis Inventory (Y-SCI) [36]; patient, parent, and clinician reports. The Y-SCI includes items derived, abridged, and adapted for adolescents from the original scale [37].
4. Strengths and Difficulties Questionnaire (SDQ) [38]: A 25-item questionnaire for assessing the psychological adjustment of children and youth, including 5 subscales—emotional symptoms, conduct problems, hyperactivity or inattention, peer-relationship problems, and prosocial behaviors.
5. Mood and Feelings Questionnaire (MFQ) [39]: A 13-item scale assessing depressive symptoms.
6. Social Adjustment Scale-Self-report (SAS-SR) [40]: A measure of social functioning in the family, school, and peer group domains.
7. Affective Reactivity Index (ARI) [41]: A 7-item parent- and self-reported concise dimensional measure of irritability.
8. Athens Insomnia Scale (AIS) [42]: An 8-item scale rated on a 0-3 scale for insomnia symptoms.
9. Sociodemographic and clinical information: Data will be collected on demographics, household composition, place of birth, parental employment, and religiosity. Information on medications, psychotherapy, ED visits, psychiatric hospitalizations due to STB, and other causes will be recorded.
10. Satisfaction survey: A 5-question (Likert-scale and open-ended) patient- and parent-satisfaction survey questionnaire about their experience and concerns about the monitoring app and procedures. A compatible, adapted version of the patient satisfaction survey will be used to capture clinicians’ experiences with ease of use and perceived utility.

### Data Analysis

#### Missing Data

While we will attempt to minimize missingness, missing data is typical and expected. Missing data will be examined for their possible clinical meaning by attrition and compliance analyses (eg, comparing completers to noncompleters and clinical correlations with missingness rate). We will investigate the pattern of missingness and use recommended methods [43] that consider the temporal nature of the data while constructing embedded, dense representations for sparse input data. We will adjust for the influence of the missingness rate on the prediction models.

#### Aim 1

We will conduct a descriptive analysis of the sample and compliance rate (mean, SD, frequency, and within-person correlations for the primary study variables). Given the expected low base rate of different suicidal behaviors, we will group the responses of the adapted C-SSRS into high-risk STB presence (Yes or No) based on their frequencies. We will assess the prediction of STB using logistic regression analyses and machine learning models (eg, random forest) for high-risk STB and nonhigh-risk STB group classification as the outcome. We will conduct multilevel modeling with a random intercept and random slope model (linear or logistic models). Predictors will be person-centered to compute within-person weekly fluctuations compared to an individual’s mean. Level 2 predictors will include the corresponding individual mean level. Sociodemographic effects and treatment intervention type and duration will be examined for their effect on the results and adjusted for. The predictive accuracy of STB by weekly surveys will be estimated by a receiver-operating characteristic curve, the generalized area under the curve, precision, recall, and *F*_1_ score [44]. We aim to identify the optimal prediction model by comparing different prediction models. The results of the satisfaction questionnaires will be analyzed using descriptive statistics and qualitative processing of open-ended text answers.

#### Aim 2

The analyses will include feature extraction, scaling, selection, embedding, and dimensionality reduction [22]. Our modeling approach uses unsupervised methods to learn useful representations using, for example, variational autoencoders [45] and underlying structures (eg, classical clustering methods). Multimodality will be handled using classical ensemble methods and the more advanced attention mechanism [46]. Similarly, sparsity will be handled using classical imputation methods and embedding (learned representation) processes [43]. Next, we will train and validate predictive models to infer higher-level activities (eg, conversation, activity and social media use) from raw sensor data. The labels will be used to train and validate the predictive models. A major challenge stems from the fact that the number of available labels is relatively small, while there is much more unlabeled (digital) data captured (continuously and passively) from the mobile phone. To use both labeled and unlabeled data, we will use semisupervised methods in conjunction with a novel method that is based on anomaly detection notions. The data will be split into training and test (holdout) data sets. Digital markers and predictive models are trained and cross-validated with k-fold cross-validation using the training set. Next, we use the test set (hold out data) to perform statistical validation and get an unbiased evaluation of the final model. The data split is stratified and performed at the patient level into 75% train and 25% test. Prediction models with STB as the outcome will adjust for potential confounders such as missingness rate, between-subject variability, diurnal and seasonal effects, and interventions. We will use classification modeling approaches (eg, random forest) appropriate for the sparsity of the data and relatively small sample size [28,47]. The performance of the machine learning methods using different data sources will be tested and compared using the generalized area under the curve, precision, recall, and *F*_1_ score [44].

### Power Statement

For multilevel models, we considered an unconditional 2-level model of the within-person longitudinal data with repeated weekly assessments (Level 1) collected across subjects (Level 2). Assuming a 75% completion rate with a power of 80%, repeated measures=25 (ie, weekly entries over 6 months), and ICC=0.5 (based on prior longitudinal studies), we need a sample of 90 adolescents. For machine learning models, the sample size represents the data held out for validation. Based on our previous studies in this population [48], over 6 months of follow-up, we expect at least 15% of the patients will have one or more self-harm incidents and 10% will have repeated ED admissions and hospitalizations. For chi-square goodness of fit, a medium effect size (*d*=0.5), and a power of 80%, 55 incidents are needed for validation, which will be approximately 10 unique patients. A Mann-Whitney-Wilcoxon test with similar power, effect size, and alpha provides similar results.

### Ethics Approval

Study procedures were approved by the Rabin Medical Center Institutional Review Board (ID #0433-20). Specific ethical considerations that were included in the study protocol are detailed below.

### Data Management and Confidentiality

The study assessment battery, including demographic information and self-reported outcome measures, will be completed through a secured web-based survey. The iFeel app ensures user privacy and anonymity with proper security data server protection software (eg, no data could be paired to a specific user). Privacy and information security comply with the European Union’s privacy requirements. The data will not include any other identifying information. The research coordinator will record the key to identifying details on a secured log, which will be kept separately and password protected.

### Adverse Events and Safety Procedures

Study assessments are performed by clinical research staff trained in suicide risk assessment using the study scales. Senior clinical staff and principal investigators will be available to the research staff by cell phone. Patients and parents will be informed that confidentiality will be kept unless there is a serious and imminent safety concern. In such an event, the disclosure will be made to a licensed clinician on the study team, the principal investigators, and the child’s therapist. The surveys will only be sent during regular business hours (8 AM-4 PM) to ensure the research team has the capacity to respond to weekly surveys that positively endorse STB. If a participant rates STB above the predefined clinical cut point, a follow-up question will be triggered to confirm their valid response. If they confirm active intent to self-harm, a text box will be prompted, providing information on emergent clinical services. Immediately following these endorsements, the principal investigators and study staff will receive an email regarding the participants’ responses. The principal investigators or licensed clinicians will thoroughly assess the participant during the same business day and respond according to the clinical practice guidelines of the medical center. In the case of adverse events (suicide attempt or other health-risk behaviors such as substance use intoxication, as well as hospitalization for suicide risk), a full report will be submitted to the institutional review board, principal investigators, and directors of the depression clinic. Our approach is consistent with expert consensus statements on ethical and safety practices for conducting digital monitoring studies with individuals at risk for suicide [49].

## Results

We expect to find longitudinal, concurrent, and prospective associations between the digital markers and self-reported and clinical assessments of STB outcomes. Successful outcomes of the proposed study will: (1) develop the ability to predict imminent STB using mobile data in a real-time, real-world setting, (2) provide an innovative and robust methodology for future studies and clinical trials to examine mental states with minimal burden and self-report bias, and (3) provide a powerful tool to identify youth at risk for suicide. The technology for this project was developed and made available for participant recruitment. Participant recruitment and follow-up started in February 2021 and are expected to be completed by 2024. The digital monitoring and clinical follow-up assessments will continue until the last participant has finished enrollment in the study. The interim data analysis began after 6 months of recruitment, and the final data analysis will be done after recruitment and follow-up assessments are completed.

## Discussion

This study aims to fill the knowledge gap in real-time predictors of STB using active and passive digital monitoring in a clinical setting. The study has 2 main objectives: (1) to demonstrate the clinical utility of weekly digital self-reports in identifying adolescents at risk for STB and (2) to develop an integrated prediction model using passive mobile sensing data for STB prediction. Based on the study hypotheses, the main anticipated findings of this study would be the significant associations between digital assessments and clinical assessments of STB, accurate classification of adolescents with high-risk STB using passive mobile usage data, and improved prediction of high-risk STB by integrating active self-reports and passive mobile sensing.

This study will contribute to the scientific and clinical efforts to understand, detect, and prevent STB in youth. While most previous studies used intensive self-reports, previous studies have also demonstrated the usefulness of predicting symptoms in patients with major depressive disorder and mood prediction in patients using machine learning algorithms with multimodal data collection from mobile devices [23-26,50]. However, this is one of the first studies to investigate proximal predictors of youth STB using real-time mobile passive sensing, including communication and activity patterns, and clinical and self-reported assessments over 6 months. Furthermore, this study will add to the limited body of literature providing results in a naturalistic setting, which is crucial to developing optimized prediction models for practical implementation in clinical settings [29].

This study has several limitations that are important to consider. As is common in intensive longitudinal studies, there are challenges in participant recruitment and retention, expected missing data due to equipment failures and nonadherence to study protocol, concerns about the power and limitations of weekly digital self-report assessments instead of daily assessments, disruptions in passive sensing information through mobile phones, psychometric validity concerns with digital self-reports, and concerns about overfitting and generalizability. To address these limitations, the study has developed a protocol for participant recruitment and retention, implemented reminder protocols and positive reinforcements, has backup systems for data, followed recommended practices for self-report reliability and validity, and will perform robust testing methodologies for working with small data sets and outcome points. The study will also adjust for mental health interventions received and document each contact.

While more intensive daily reports could better capture STB fluctuations and associations, we believe weekly assessments will be novel, feasible, and most generalizable for clinical settings. Moreover, the substantial reliance on daily self-reports requires greater compliance, which could burden patients, and weekly digital assessments of STB rather than daily could lead to greater satisfactory compliance, which can be translated to clinical practice. The goal of the study is to provide proof of concept for using digital technology through mobile phones to identify the risk of youth suicidal behaviors more effectively. The results of this study will be the first step toward large-scale validation that may ultimately result in practical prediction models and immediate interventions to prevent suicide.

The results of this study can have a significant impact on developing future technology-based projects for suicide prevention. To ensure that the findings reach a wider audience, we plan to disseminate the study results through multiple channels, including manuscripts, social media, webinars, and conferences. The aim is to reach a broad audience of health care professionals, researchers, and policy makers. By disseminating the study’s results through multiple channels, we hope to maximize its impact and ensure that it reaches a broad audience, including clinicians, researchers, policy makers, and advocacy groups. Future investigations have the potential to expand upon the findings of this study and contribute to the development of more advanced prediction models for STB in adolescents. Due to the relatively rare occurrence of suicidal behaviors, it may be beneficial to conduct a large-scale study or engage in collaborative research efforts focused on digital monitoring and prediction of STB. In addition, by combining physiological measurements obtained through wearable devices, valuable information on activity and arousal can be incorporated, validating the smartphone-based digital markers and addressing certain limitations. Therefore, the combination of these approaches in future studies offers advantages. Moreover, future advancements may involve the inclusion of additional data sources such as acoustic features and natural language processing, which were not considered in this particular study. By expanding the scope of data collection, a more comprehensive understanding of STB prediction can be achieved. Finally, to accomplish the goal of prevention, future studies should also concentrate on developing and evaluating adaptive interventions that take into account individual circumstances indicated by digital monitoring. These interventions should be informed by data obtained through both active and passive monitoring, allowing for effective responses to digital prompts and the provision of timely support.

Defining proximal risk factors in youth may significantly improve the clinical evaluation of imminent suicide risk. Relying more on passive sensing than self-reported data to identify the risk for suicidal behavior will make interventions more scalable and effective. With minimal assessment burden and reliance on patient disclosure of suicidal intent, digital markers can inform different interventions and provide an objective means to measure psychosocial or pharmacological treatment efficacy. Integrating multiple self-reported and passive data sources into a validated prediction model can improve the identification of adolescents at risk for STB. The ability to predict STB using mobile data in real-time, real-world settings has tremendous potential for suicide risk identification and prevention. It can enable personalized and just-in-time interventions triggered based on passive mobile usage and self-reported data, preventing adolescent injury and mortality.

## Abbreviations

ED: emergency department
STB: suicidal thoughts and behavior

## Data Availability

The data sets that will be generated or analyzed during the study can be obtained from the corresponding author on reasonable request.
